# Cardio-Renal Syndrome: Latest Developments in Device-Based Therapy

**DOI:** 10.3390/jcm13247814

**Published:** 2024-12-20

**Authors:** Vlad Meche, Nilima Rajpal Kundnani, Abhinav Sharma, Flavia-Maria Căpăstraru, Daciana Nistor, Cristian Andrei Sarau, Laura Gaita

**Affiliations:** 1 Doctoral School, Faculty of Medicine, “Victor Babes” University of Medicine and Pharmacy, 3000041 Timisoara, Romania; 2 University Clinic of Internal Medicine and Ambulatory Care, Prevention and Cardiovascular Recovery, Department VI—Cardiology, “Victor Babes” University of Medicine and Pharmacy, 3000041 Timisoara, Romania; knilima@umft.ro (N.R.K.); sharma.abhinav@umft.ro (A.S.); 3 Research Centre of Timisoara Institute of Cardiovascular Diseases, “Victor Babes” University of Medicine and Pharmacy, 3000041 Timisoara, Romania; 4 Faculty of Medicine, “Victor Babes” University of Medicine and Pharmacy, 3000041 Timisoara, Romania; 5 Department of Functional Sciences, Physiology, Center of Immuno-Physiology and Biotechnologies (CIFBIOTEH), “Victor Babes” University of Medicine and Pharmacy, 300041 Timisoara, Romania; 6 Centre for Gene and Cellular Therapies in Cancer, 300723 Timisoara, Romania; 7 Department of Medical Semiology I, “Victor Babes” University of Medicine and Pharmacy, 300041 Timişoara, Romania; 8 Municipality University Emergency Hospital, 300254 Timisoara, Romania; 9 Second Department of Internal Medicine, “Victor Babes” University of Medicine and Pharmacy, 300041 Timisoara, Romania; gaita.laura@umft.ro; 10 “Pius Brînzeu” Emergency County Hospital, 300723 Timisoara, Romania

**Keywords:** cardio-renal syndrome (CRS), Acute Dialysis Quality Initiative (ADQI), heart failure, kidney damage, devices

## Abstract

**Background:** Cardio-renal syndrome (CRS) is a complex condition involving bidirectional dysfunction of the heart and kidneys, in which the failure of one organ exacerbates failure in the other. Traditional pharmacologic treatments are often insufficient to manage the hemodynamic and neurohormonal abnormalities underlying CRS, especially in cases resistant to standard therapies. Device-based therapies have emerged as a promising adjunct or alternative approach, offering targeted intervention to relieve congestion, improve renal perfusion, and modulate hemodynamics. This study aimed to evaluate the efficacy and safety of various device-based therapies in CRS management, utilizing DRI2P2S classification to categorize interventions as dilators, reducers, interstitial modulators, pullers, pushers, and shifters. **Methods:** A comprehensive analysis of clinical trial data and observational studies involving device-based therapies in patients with CRS was conducted, with a focus on hemodynamic endpoints, renal and cardiac function, symptom relief, and adverse events. Devices included in the analysis were splanchnic denervation systems (dilators), devices for central and pulmonary pressure reduction (reducers), and systems targeting interstitial fluid (fluid shifters), among others. A systematic literature review from 2004 to 2024 was performed using databases including PubMed, Embase, and ClinicalTrials.gov, following PRISMA guidelines for study selection. Data were extracted on patient demographics, device type, trial design, outcomes, and follow-up duration. **Results:** Device-based therapies demonstrated varying levels of efficacy in CRS, with significant improvements observed in specific parameters. Notable results were a reduction in central venous pressure and improved diuretic responsiveness in acute CRS cases, while also stabilizing or improving renal function. Other relevant endpoints were fewer heart failure hospitalizations and a reduction in renal adverse events, reduced tissue congestion and improved quality of life scores. However, some devices presented challenges, including procedure-related complications and a learning curve for optimal device implantation. **Conclusions:** Device-based therapies offer a valuable addition to the CRS treatment paradigm, particularly in cases unresponsive to conventional diuretics and other pharmacologic measures. Each of them addresses specific pathophysiological components of CRS and shows promise in improving clinical outcomes. Nevertheless, further large-scale, long-term trials with comprehensive endpoints are needed to establish these therapies’ roles in standard care and to optimize patient selection criteria. Enhanced understanding of device mechanisms and refinement of trial endpoints will be key to maximizing the impact of these therapies on quality of life and clinical outcomes for CRS patients.

## 1. Introduction

Cardio-renal syndrome is a complex disease that requires treatment of both the renal and cardio-vascular systems. The usual course of medical treatment nowadays is to care for one system primarily (the one which caused the complex activation of this “vicious cycle” of physiopathology, which is more often than not difficult in clinical practice) and to act with caution so as not to damage the other one too much. Practice and theory have both shown that “forward failure” and “backward failure” of both the heart and the kidneys exist, which requires a multi-faceted view of this syndrome, along with a tight collaboration between cardiology and nephrology.

Therefore, the need for a bilateral approach, with minimally invasive devices, which work in different manners (by retrograde activation of the “unstressed” venous pooled blood, or by anterograde mobilization of the arterial blood, or by shifting the interstitial fluid through the lymphatic circulation and back into the main venous tracts), has been increasingly tested and shows promise for the future.

Devices such as these are mostly in incipient clinical trial phases; thus, the purpose of this study was to describe their classification, their purpose, and the latest studies supporting their usage, but also to elaborate on any limitations that might have to be overcome when implementing them in standard heart failure and kidney damage care simultaneously.

### 1.1. Overview of Cardio-Renal Syndrome

Cardio-renal syndrome (CRS) refers to a spectrum of disorders in which either acute or chronic dysfunction in the heart or kidneys induces acute or chronic dysfunction in the other organ. Because of the complex interplay between the heart and kidneys, CRS is subdivided into five distinct classifications (types 1–5) based on which organ is the initial driver and the chronicity of the conditions involved [1]. This classification system, developed by the Acute Dialysis Quality Initiative (ADQI), aids in understanding the bidirectional impact of these systems. Therefore, the five types are as follows: type 1 CRS (acute cardio-renal syndrome), where acute heart failure leads to acute kidney injury (AKI); type 2 CRS (chronic cardio-renal syndrome), where chronic heart dysfunction progressively worsens kidney function; type 3 CRS (acute reno-cardiac syndrome), where acute kidney injury precipitates acute heart failure; type 4 CRS (chronic reno-cardiac syndrome), where chronic kidney disease (CKD) contributes to worsening of the chronic heart dysfunction; and type 5 CRS (secondary cardio-renal syndrome), where a systemic condition (such as sepsis or amyloidosis) simultaneously affects both organs, leading to dysfunction [2].

It should be noted that CRS is distinct from acute decompensated heart failure (ADHF) and isolated renal dysfunction. In ADHF, fluid overload and increased pressure lead to cardiac output issues, but renal impacts are often secondary and less systemic. In contrast, worsening renal failure typically lacks the bidirectional impact of CRS. It rather involves isolated kidney impairment without substantial cardiac complications at onset. CRS, by contrast, involves bidirectional pathology where both the heart and kidneys are directly impaired in tandem, similar to a “vicious cycle”, necessitating a more nuanced approach to treatment [3].

The primary physio-pathological mechanisms underlying CRS involve, among others:

- Hemodynamic factors such as increased venous congestion and reduced renal perfusion from elevated central venous pressures, which often accompany heart failure.
- Neurohormonal dysregulation, stemming from activation of the renin–angiotensin–aldosterone system (RAAS) and the sympathetic nervous system, causing further renal vasoconstriction and sodium retention.
- Inflammatory pathways are activated in both organs, promoting fibrotic changes and vascular remodeling.
- Oxidative stress contributes to endothelial dysfunction, exacerbating damage in both cardiac and renal tissues.

As mentioned previously, although a rise in serum creatinine in the context of acute heart failure (AHF) is often assumed to indicate CRS, not every creatinine increase signals a true cardio-renal syndrome. Creatinine fluctuations can originate from several other factors unrelated to direct kidney injury or dysfunction, such as hemodynamic changes, medication effects, or transient shifts in fluid status. Understanding the nuances between true CRS and other causes of elevated creatinine is crucial to guide appropriate treatment decisions in AHF [4].

### 1.2. Diuretic Resistance in Cardio-Renal Syndrome

Diuretic resistance, defined as a diminished or absent response to diuretic therapy, is a common and challenging issue in managing CRS, particularly in type 1 CRS. Patients with AHF and CRS may initially respond to loop diuretics but eventually require higher doses or additional diuretics to achieve the same level of fluid removal. This resistance can result from multiple mechanisms, including sodium retention, altered renal handling of diuretics, or changes in the neurohormonal axis, which lead to decreased natriuresis (sodium excretion) [5].

Diuretic resistance often arises due to “braking” mechanisms that the body deploys in response to chronic diuretic use, aiming to conserve sodium and water. This adaptive response, combined with reduced renal perfusion in CRS, results in the kidney retaining sodium despite high-dose diuretic administration [6].

It should be noted that diuretic resistance is not synonymous with CRS, and that the device-based therapies included in this review are described for both states.

### 1.3. Sodium Avidity in CRS

“Sodium avidity” is a term used to describe the kidney’s tendency to reabsorb sodium aggressively, often in response to neurohormonal activation, such as increased levels of angiotensin II, aldosterone, and sympathetic nervous activity, which are common in CRS [7]. In a state of sodium avidity, even when patients receive loop diuretics, the kidneys may retain sodium in the distal nephron segments, which limits the efficacy of diuretics. Sodium avidity is particularly problematic in CRS because the kidneys respond as if they are in a volume-depleted state, despite the presence of extracellular fluid overload. This leads to persistent fluid retention and edema, despite the use of potent diuretics.

Resistance to diuretic therapy due to sodium avidity is often compounded by high central venous pressures in CRS, which further reduces renal blood flow, impairs diuretic delivery to the nephron, and intensifies sodium retention.

This mechanism is clinically relevant, as multiple studies have shown a correlation between low urine sodium concentration and worse outcomes for the patient. For instance, in the ROSE-AHF trial [8], which evaluated 360 patients with acute heart failure and renal dysfunction, a lower urinary sodium in the first 24 h identified patients at risk for longer hospitalization. Moreover, Hoen et al. [9] found a correlation in 143 patients admitted for ADHF between lower spot urinary sodium and more severe cardio-renal disease, but also found that spot urinary sodium predicts 90-day HF hospital-free survival in ADHF. When it comes to long-term survival, however, Ganes et al. [10] evaluated 188 men with chronic HF and found that urinary sodium was a significant predictor of all-cause mortality but not MACE outcomes over 28–33 years, with 173–229 mmol/day appearing to be the optimal level.

### 1.4. The Concept and Utility of a “Diuretic Holiday”

A “diuretic holiday” refers to a temporary pause or reduction in diuretic therapy, typically with the aim of resetting the kidney’s response to diuretics, reducing neurohormonal activation, and minimizing sodium avidity. Diuretic holidays may help restore the kidneys’ responsiveness to diuretics, especially in patients with CRS and diuretic resistance [6].

By reducing or pausing diuretic doses for a period, clinicians allow the kidneys to “reset” and mitigate the braking response, potentially improving subsequent diuretic efficacy. During a diuretic holiday, alternative management strategies, such as careful volume monitoring and adjustments to dietary sodium intake, can support fluid balance.

### 1.5. Device-Based Therapy in CRS Management

Device-based therapies for CRS have emerged to manage the bidirectional dysfunction in a targeted manner. Despite the promising results showed by more recent decongestive therapy in an ADHF context, such as acetazolamide in the ADVOR [11] trial, the association of loop diuretics with thiazidic in CLOROTIC [12], or finerenone in studies such as FINE-ARTS [13], conventional pharmacological therapies often become insufficient or exacerbate renal injury in patients with CRS. Hence, device-based therapies could prove to be more effective in managing fluid overload and enhancing cardiac output or renal function without the adverse effects of medications. Devices such as ultrafiltration systems, percutaneous mechanical circulatory support devices (e.g., Impella, intra-aortic balloon pumps), and implantable hemodynamic monitors are currently being utilized to manage both cardiac and renal complications in CRS [14].

Ultrafiltration, for example, allows precise fluid removal, helping reduce central venous pressure and improve cardiac preload without causing drastic drops in renal perfusion, which is crucial in CRS type 1 and type 2 [15,16]. Similarly, mechanical circulatory support devices can help maintain systemic perfusion pressures, supporting both cardiac and renal functions.

### 1.6. Forward Failure, Backward Failure, and DRI2P2S Classification

In cardio-renal syndrome, understanding specific mechanisms that contribute to kidney dysfunction is critical for developing effective treatments. The concepts of “forward failure” and “backward failure” of the kidneys provide a useful framework for understanding how cardiovascular dysfunction translates into renal pathology.

Forward Failure of the Kidney: This concept, also referred to as “prerenal failure”, occurs due to reduced perfusion pressure from decreased cardiac output [17]. In the context of heart failure, diminished blood flow to the kidneys leads to a decreased glomerular filtration rate (GFR) and may ultimately cause renal hypoxia. This decreased renal perfusion is often exacerbated by vasoconstriction mechanisms, such as activation of the renin–angiotensin–aldosterone system (RAAS), which attempts to maintain blood pressure but can reduce renal blood flow further.

Backward Failure of the Kidney: Also known as “congestive renal failure”, backward failure occurs when elevated venous pressure from right-sided heart failure leads to kidney congestion [18]. Increased central venous pressure reduces the pressure gradient required for renal blood flow and leads to venous congestion, interstitial edema, and compromised GFR. This backward pressure also contributes to renal parenchymal injury over time [19].

These two mechanisms operate together in many cases of CRS, especially in advanced heart failure, creating a scenario where low forward perfusion pressure and high backward venous pressure each drive renal impairment.

### 1.7. DRI2P2S Classification and Pathophysiological Mechanisms in CRS

The DRI2P2S classification [6] offers a structured way to understand CRS-related mechanisms by categorizing them as “drivers” (factors that lead to renal failure) and “modifiers” (factors that worsen or modify the progression of CRS).

The DRI2P2S classification system categorizes therapeutic interventions in cardio-renal syndrome (CRS) based on their mechanisms of action in managing fluid balance and circulatory dynamics. The acronym DRI2P2S stands for:

1. D—Dilators (splanchnic denervation) or Decongestive (diuretics, aquapheresis, peritoneal dialysis).
2. R—Renal Replacement (CRRT-continuous renal replacement therapy, peritoneal dialysis)—AlfaPump, Reprieve System.
3. I(1)—Inotropes (cardiac plexus nerve stimulation)—Cardionomic, NeuroTronik.
4. I(2)—Interstitial (fluid management: lymphatic duct compression)—WhiteSwell.
5. P—Pullers (suprarenal IVC blood pump, intrarenal vein pump, infrarenal partial obstruction, intermittent SVC occlusion)—preCardia, Doraya catheter, transcatheter renal decongestion system.
6. P—Pushers (suprarenal descending aortic pumps)—Reitan catheter pump, Aortix, Second Heart Assist.
7. S—Selective (selective intrarenal artery vasodilator drug delivery)—Benephit catheter.

Each category targets a specific aspect of the complex interplay between the heart and kidneys in CRS. Below are the details of each category and examples of therapies and devices that fall under each classification, with the latest approved pilot studies, animal models, or human studies.

## 2. Decongestive (D)

Decongestive therapies are aimed at reducing fluid overload and venous congestion, which are hallmarks of CRS. This category primarily includes pharmacological and device-based diuretics, as well as other fluid-removal techniques that alleviate systemic and pulmonary congestion, reduce cardiac workload, and improve kidney function by lowering venous pressures.

- Loop Diuretics (e.g., furosemide, torasemide) are often first-line therapies to promote diuresis and decrease fluid overload. Trials like ADVOR [11] (acetazolamide) or CLOROTIC [12] (combination of loop diuretics with thiazidic) have proven to relieve congestion to a somewhat greater extent, but without significant impact on mortality and readmission for heart failure.
- Aquapheresis is a form of ultrafiltration therapy that removes excess fluid directly from the bloodstream, offering precise control of fluid removal in cases of diuretic resistance. As the AVOID-HF [20] trial, in which aquapheresis was compared with IV diuretics and hospitalization, showed, the aquapheresis group trended toward a longer time to first HF event within 90 days and fewer HF and cardiovascular events. However, more patients in the aquapheresis group experienced adverse events of special interest or serious product-related adverse events, with similar renal function changes in both arms.

Other sources in the literature view the acronym “D” as standing for “Dilators”, which are devices or techniques designed to improve blood flow and reduce vascular resistance by actively dilating blood vessels. By reducing systemic and regional vascular resistance, these devices can alleviate the burden on the heart, improve renal perfusion, and facilitate better fluid management, addressing both forward and backward failures in CRS [21]. One notable trial studying this matter regarding medication which might work as a “dilator” is the ROSE trial [22], which studied the dose-dependent effect of dopamine and nesitiride on dilating renal arteries. In total, 360 patients with AHF and renal dysfunction were randomized to receive placebo, low-dose dopamine, or low-dose nesitiride. Neither low-dose dopamine nor low-dose nesiritide enhanced decongestion or improved renal function when added to diuretic therapy, which stresses again the need for newer, device-based therapies for effective decongestion through “dilation”.

One emerging and innovative approach under this category is splanchnic nerve blockade or splanchnic denervation [23]. This method targets the splanchnic nerves that regulate blood flow in the abdominal organs. The splanchnic vasculature can store up to 20% of the body’s blood volume, so, by reducing vasoconstriction in this region, splanchnic denervation helps redistribute blood away from the overloaded heart and pulmonary circulation, thus reducing central venous pressure (CVP) and enhancing cardiac and renal function [24,25]. Fudim et al. [23] conducted a study of splanchnic nerve modulation in patients with acute decompensated heart failure and CRS. The study observed that “splanchnic nerve blockade significantly reduced central venous pressure and pulmonary capillary wedge pressure, resulting in decreased symptoms of congestion and improved cardiac output without a drop in systemic blood pressure”. Patients also experienced improved renal perfusion, evidenced by modest improvements in the glomerular filtration rate (GFR) over a 6-month follow-up.

In a cohort of patients with chronic CRS (type 2), Fudim et al. [26] examined the use of splanchnic nerve blockade over a 12-month period. The authors found that “long-term splanchnic nerve blockade maintained reduced central venous pressure, leading to sustained reductions in edema and hospitalizations due to heart failure exacerbation”. This long-term effect on CVP was associated with fewer episodes of worsening renal function, suggesting that sustained relief from congestion could help slow CRS progression.

A trial conducted in 2022 [27] investigated the effect of splanchnic nerve ablation on refractory CRS cases characterized by chronic fluid overload unresponsive to high-dose diuretics. The study reported that “splanchnic ablation provided a significant reduction in diuretic requirement by decreasing venous congestion and enabling greater redistribution of blood volume into the splanchnic bed”. Additionally, patients had fewer hospitalizations and showed improved renal function markers at 1-year follow-up, indicating that splanchnic ablation could serve as an adjunct to pharmacotherapy in diuretic-resistant CRS patients.

## 3. Renal Replacement (R)

Renal replacement therapy (RRT) is used in cases where kidney function is severely impaired and conservative measures are insufficient to manage fluid and electrolyte balance. This is especially relevant in advanced CRS cases where fluid overload and waste products cannot be adequately removed by the kidneys.

It should be noted that ultrafiltration and renal replacement therapy are two similar methods to obtain decongestion, but also that ultrafiltration is actually a sub-type of renal replacement therapy. This nomenclature difference makes it easy to confound the two, which is why it is important to understand the mechanisms behind them. The main goal of ultrafiltration is to remove fluid using semipermeable membranes without volume replacement, thus achieving volume but not solute control in the patient [28,29]. “Renal replacement therapy” (RRT) is an umbrella-term which includes ultrafiltration, but also hemodialysis, hemofiltration, hemodiafiltration, plasmapheresis, and hemoperfusion, and it refers to any type of therapy used to replace the normal blood-filtering function of the kidneys.

- Continuous Renal Replacement Therapy (CRRT) is often used in critically ill CRS patients to provide continuous fluid and solute removal without destabilizing blood pressure. This method is preferable for patients who are hemodynamically unstable [30].
- Intermittent Hemodialysis can be used for patients with less severe CRS who can tolerate the more rapid fluid and solute shifts [31].

## 4. Inotropes (I1)

Inotropes increase cardiac contractility and help to improve cardiac output, thus enhancing renal perfusion. Inotropic support is especially relevant in low-output CRS, where poor cardiac output results in inadequate renal perfusion and subsequent kidney dysfunction.

- Dobutamine is a common inotropic agent used to enhance cardiac output by stimulating beta-adrenergic receptors. However, it is generally reserved for acute decompensations, due to its arrhythmogenic potential [32].
- Milrinone is a phosphodiesterase-3 inhibitor that has both inotropic and vasodilatory effects, making it useful for patients with low cardiac output and high systemic vascular resistance [33].
- Cardiac plexus nerve stimulation has recently been proven useful in treating patients with type 2 cardio-renal syndrome [34].

It is important to take into account, however, that inotropes should be reserved for acute and grave situations only, and that they do not decrease mortality in patients presenting with cardio-renal syndrome. As a meta-analysis from 2021 including data from 37 RCTs studying patients with advanced heart failure requiring inotropic medication has shown [35], levosimendan (an inodilatator and ATP-dependent potassium channel activator) was the only inotrope which could statistically reduce mortality and increase GFR in patients with HF. However, the statistics of the GFR values could not be interpreted as having clinical significance. All the other inotropes either had no effect on or increased mortality over time. This shows again the driving need for device-based therapies for improving cardiac output, since current inotropic medication is definitely not ideal.

## 5. Interstitial Fluid Management (I2)

Interstitial fluid management targets the lymphatic system and fluid exchange between capillaries and the interstitial space. By promoting efficient fluid shift from the interstitium back to the circulation or directly removing interstitial fluid, therapies in this category help alleviate tissue edema and systemic congestion.

- WhiteSwell eLym [36] stimulates lymphatic flow to help clear interstitial fluid and reduce central venous congestion.
- AquaPASS [37] promotes peritoneal absorption of excess fluid, gradually reducing congestion without diuretic medications.

## 6. Pullers (P1)

Pullers are devices or methods that target venous circulation, reducing congestion by improving venous return to the heart or decreasing central venous pressure (CVP) [38]. This category addresses the “backward failure” phenomenon in CRS, where elevated CVP impedes kidney perfusion [18].

- The Doraya Catheter [39] is a temporary catheter placed in the inferior vena cava that reduces CVP by modulating venous return to the right heart, improving renal perfusion.
- The preCARDIA System [7] applies gentle pressure to the vena cava to alleviate venous congestion and improve kidney function.

## 7. Pushers (P2)

Pushers are therapies that enhance arterial flow and increase renal perfusion pressure, helping to address “forward failure” in CRS. By improving systemic blood flow, these devices can ensure adequate perfusion of the kidneys, especially in patients with low cardiac output [40].

- Aortix [41] is an intra-aortic device that propels blood forward during diastole to enhance renal perfusion without increasing cardiac workload.
- ModulHeart [42] is a modular support device placed in the aorta to optimize systemic perfusion and directly improve blood flow to the kidneys.

## 8. Sympatholytics (S)

Sympatholytic agents reduce sympathetic nervous system (SNS) activity, which is frequently overactivated in CRS. This overactivity leads to increased systemic vascular resistance, fluid retention, and progression of renal dysfunction. By reducing SNS activity, sympatholytics help improve both cardiac and renal function.

- Beta-blockers (e.g., carvedilol) are used to inhibit sympathetic activity, reducing heart rate and decreasing myocardial oxygen demand. This allows the heart to pump more efficiently, reducing stress on both the heart and kidneys [43].

The classification includes “pushers”, “pullers”, and “fluid shifters”, referring to hemodynamic, vascular, and fluid-related forces, respectively, influencing renal function.

Pushers: These mechanisms involve forces that increase renal congestion or venous pressures [44]. Elevated venous pressure, especially from right-sided heart failure, pushes fluid back toward the kidneys, leading to venous congestion. This congestion reduces the filtration gradient and, consequently, the GFR, promoting fluid overload. RAAS activation exacerbates this by causing vasoconstriction, worsening renal congestion [45] (Table 1).

Pullers: Pullers refer to mechanisms that decrease effective circulatory volume, pulling away blood flow from the kidneys and compromising perfusion. Reduced cardiac output (forward failure) and excessive diuresis are common “pulling” mechanisms in CRS. Hypoperfusion triggers compensatory mechanisms like RAAS and sympathetic nervous system activation, further decreasing renal perfusion and contributing to ischemic injury [46].

Fluid Shifters: These mechanisms involve shifts in body fluid compartments, altering intravascular, interstitial, and intracellular fluid distribution. In CRS, interstitial edema due to fluid retention and systemic inflammation shifts fluid into the kidney’s interstitial space, causing renal congestion and impairing tubular function. Additionally, increased interstitial fluid leads to fibrosis over time, reducing the kidney’s ability to respond to hemodynamic shifts [47,48].

The combination of these “pushers”, “pullers”, and “fluid shifters” leads to a scenario in which both hemodynamic and humoral factors create a hostile environment for the kidneys, promoting progressive dysfunction. These factors also generate a feedback loop that exacerbates both cardiac and renal impairments.

### 8.1. “Pullers” in Cardio-Renal Syndrome

“Pullers” in cardio-renal syndrome (CRS) refer to mechanisms and therapeutic interventions that address the challenge of reduced effective circulatory volume and renal hypoperfusion [44]. In CRS, impaired cardiac function results in inadequate forward flow and perfusion to the kidneys, exacerbating renal dysfunction [7]. Addressing venous congestion has emerged as a primary therapeutic target, and devices such as the Doraya catheter, preCARDIA system, and Perfusor device are designed to optimize venous return and alleviate renal congestion in CRS patients [21,49].

### 8.2. Venous Circulation in CRS

In CRS, venous congestion significantly contributes to renal impairment. Elevated central venous pressure (CVP), often a result of right-sided heart failure, impedes effective renal perfusion. High venous pressures create a “backward” force, elevating renal interstitial pressure, which reduces the gradient for glomerular filtration and diminishes the glomerular filtration rate (GFR) [50]. Moreover, increased CVP leads to venous congestion, causing renal parenchymal edema and subsequently damaging renal structures such as the tubules, glomeruli, and vasculature. This mechanism of “backward failure” is compounded by a reduced effective circulating volume, contributing to a reduction in renal blood flow and oxygenation [51].

The venous congestion associated with CRS also stimulates maladaptive neurohormonal pathways, including the renin–angiotensin–aldosterone system (RAAS) and sympathetic nervous system (SNS), which attempt to compensate for reduced perfusion but lead to vasoconstriction and sodium retention [52]. These adaptive responses, though intended to maintain blood pressure and perfusion, ultimately exacerbate renal congestion and fluid overload [53].

Key studies have demonstrated that reducing venous congestion improves renal function in CRS patients [54,55]. By reducing CVP and renal venous pressure, “pullers” such as device-based therapies offer an alternative to diuretics, which often become less effective or contribute to electrolyte imbalances and renal impairment in advanced heart failure [56,57].

### 8.3. Device-Based Therapies for Venous Congestion in CRS

#### 8.3.1. The Doraya Catheter

**Figure 1:**
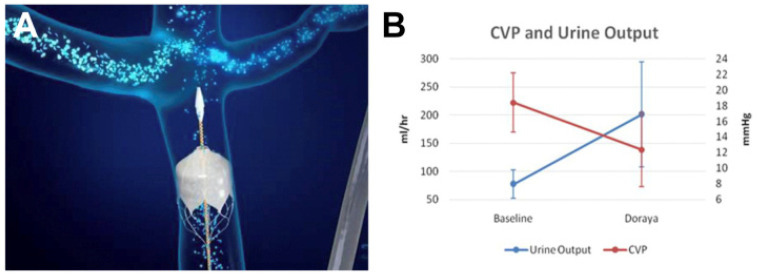
The location of the catheter in the inferior vena cava and its clinical effects. (**A**) The location of the catheter in the inferior vena cava, and (**B**) improvement in the mean central venous pressure (CVP) and urine output values following catheter placement [58].

The Doraya catheter is designed to alleviate renal congestion by creating controlled stenosis in the inferior vena cava (IVC) below the renal veins. By partially occluding the IVC, this catheter selectively reduces venous congestion in the kidneys without impacting overall venous return from other organs. The device functions by balancing the intrarenal venous pressure, and thus improves renal perfusion without inducing systemic hypotension [49] (Figure 1).

One of the primary trials of the Doraya catheter demonstrated that it effectively reduces renal venous congestion and improves renal function markers in CRS patients. In the study, Zymliński et al. [58] showed that CRS patients treated with the Doraya catheter (developed by Revamp medical) had a reduction in central venous pressure, improved renal filtration, and decreased need for diuretic use compared to controls who did not receive catheter-based intervention. The study included seven males and two females, and the inclusion criteria comprised patients with fluid overload symptoms, insufficient diuretic response, N-terminal pro–B-type natriuretic peptide ≥ 1600 pg/mL, and central venous pressure (CVP) ≥ 12 mm Hg. The catheter device was deployed for up to 12 h. The primary safety outcome was the rate and nature of device- and procedure-related AEs until 1 month following the procedure. The catheter created a transient and fully controllable gradient in the IVC. Its deployment resulted in significant pressure reduction above the device of 12.4 ± 4.7 mm Hg, compared to unchanged pressure below the catheter (18.5 ± 6.2 mm Hg) at the end of procedure (*p* < 0.01; by Student’s *t*-test for paired samples).

The rate of diuresis was measured as 77.1 ± 25 mL/h at baseline and 200.8 ± 93 mL/h during device deployment, while maintaining the same diuretic dose. Furthermore, the average peak urine output rate during deployment was 294 ± 139 mL/h.

A positive natriuretic signal also existed during the procedure; however, the spot urine sodium was measured only in a limited number of patients (n = 3), and the mean increased from 35 to 101 mmol/L. The clinical signs of congestion, namely edema and dyspnea, improved from baseline (1.8 ± 0.8 and −1.4 ± 1.1, respectively) to the postprocedural period (0.7 ± 0.9 and 1.1 ± 0.9, respectively).

This improvement in kidney and heart function was attributed to the reduction of venous congestion directly impacting renal perfusion pressure. Although the study was not designed to investigate the clinical efficacy of the catheter, early positive signs suggest potential for future development, and it may be speculated that this device may be beneficial in patients suffering from an inadequate diuretic response.

#### 8.3.2. The preCARDIA System

**Figure 2:**
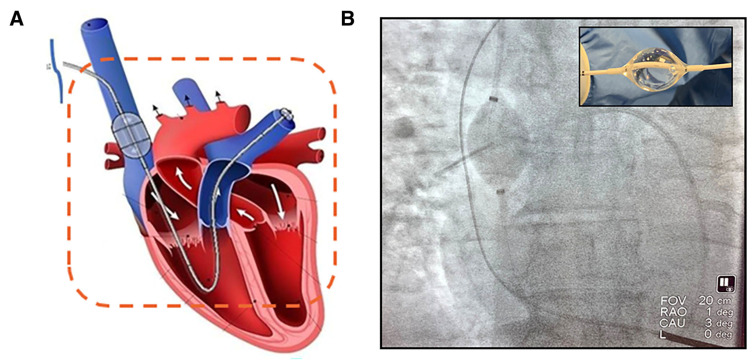
The preCARDIA system. (**A**) An illustration of the balloon-mounted catheter and pump with console designed to intermittently occlude the superior vena cava (SVC) by infusing small volumes of saline using a controlled duty-cycle timing algorithm. Arrows show the normal flow of blood through the right and left heart respectively. (**B**) Fluoroscopic image of the preCARDIA catheter in a patient. The inset image shows the SVC occlusion balloon. CAU, caudal; FOV, frame of view; L, left; and RAO, right anterior oblique [7].

The preCARDIA system is an extracorporeal device designed to intermittently occlude the superior vena cava (SVC), reducing preload and effectively managing fluid overload in heart failure patients. This device offers temporary unloading of the venous system, which can help mitigate renal congestion and improve renal function by enhancing forward flow (Figure 2).

The VENUS-HF Trial [7], a study of the preCARDIA system, evaluated its impact on hemodynamic parameters and kidney function in CRS patients. In a multicenter, prospective, single-arm exploratory safety and feasibility trial, 30 patients with acutely decompensated heart failure were assigned to preCARDIA therapy for 12 or 24 h. The primary safety outcome was a composite of major adverse cardiovascular and cerebrovascular events through 30 days. Secondary endpoints included technical success, defined as successful preCARDIA placement, treatment, and removal and reduction in right atrial and pulmonary capillary wedge pressures. The results showed a significant reduction in right atrial pressure (RAP) and CVP, as well as improved patient-reported symptoms and functional status over 72 h of treatment (right atrial pressure decreased by 34% (17 ± 4 versus 11 ± 5 mm Hg, *p* < 0.001) and pulmonary capillary wedge pressure decreased by 27% (31 ± 8 versus 22 ± 9 mm Hg, *p* < 0.001); compared with pretreatment values, urine output and net fluid balance increased by 130% and 156%, respectively, with up to 24 h of treatment (*p* < 0.01)). According to Kapur et al. (authors of the VENUS-HF trial), this device showed promise in reducing the need for intravenous diuretics in CRS patients, suggesting it could be a viable non-pharmacologic strategy to manage venous congestion in heart failure with concurrent kidney dysfunction.

#### 8.3.3. The Perfusor Device

The Perfusor device targets hemodynamic stabilization by enabling precise administration of vasodilators or inotropes, which improve cardiac output and reduce venous congestion without causing hypotension. In CRS patients with reduced renal perfusion, this device helps maintain steady blood flow through the kidneys, thereby preventing renal ischemia and congestion.

Studies on the Perfusor device in CRS patients indicate that it enhances renal blood flow, prevents congestion, and decreases fluid overload. Krum et al. [59] assessed the Perfusor’s impact on renal function by administering low doses of inotropes to improve renal perfusion. Patients using the Perfusor had reduced serum creatinine levels and improved urine output, demonstrating the device’s ability to improve renal function and maintain hemodynamic stability in advanced heart failure cases.

### 8.4. “Pushers” in Cardio-Renal Syndrome

In cardio-renal syndrome (CRS), devices categorized as “pushers” are designed to enhance arterial circulation and improve renal perfusion, addressing the diminished forward flow that is characteristic of CRS. Poor arterial flow results in renal hypoperfusion, which exacerbates kidney dysfunction and contributes to the decline of both cardiac and renal function. By enhancing systemic circulation and boosting cardiac output, “pushers” aim to restore renal perfusion and mitigate the vicious cycle of CRS [60].

### 8.5. Arterial Circulation in CRS

Arterial circulation is critical for maintaining renal perfusion and glomerular filtration. In CRS, impaired cardiac output leads to inadequate arterial blood flow to the kidneys, which decreases the glomerular filtration rate (GFR) and promotes ischemic injury. This low forward flow initiates a cascade of compensatory mechanisms, such as activation of the renin–angiotensin–aldosterone system (RAAS) and sympathetic nervous system (SNS), which attempt to restore blood pressure and volume but often lead to further vasoconstriction and fluid retention [61]. These adaptations can lead to increased systemic vascular resistance, worsening heart function and further compromising kidney function.

A lack of adequate renal perfusion also leads to tubulointerstitial hypoxia and fibrosis over time, damaging renal structures and further reducing GFR [62]. In this context, devices that enhance arterial flow, referred to as “pushers”, can improve renal perfusion pressure, alleviate hypoxic injury, and break the cycle of worsening CRS [40].

### 8.6. Device-Based Therapies for Arterial Circulation in CRS

Several innovative devices aim to boost arterial flow to the kidneys, effectively “pushing” blood forward to improve renal perfusion and mitigate the impact of low cardiac output on renal function [63].

#### 8.6.1. The Reitan Catheter Pump

The Reitan Catheter Pump is an intra-aortic device that increases cardiac output by augmenting blood flow during diastole. It is positioned in the descending aorta, where it intermittently propels blood forward, creating a pulsatile flow that enhances renal perfusion without causing an increase in myocardial oxygen demand. This design also reduces afterload, assisting the heart in maintaining output without excessive strain [64].

**Figure 3:**
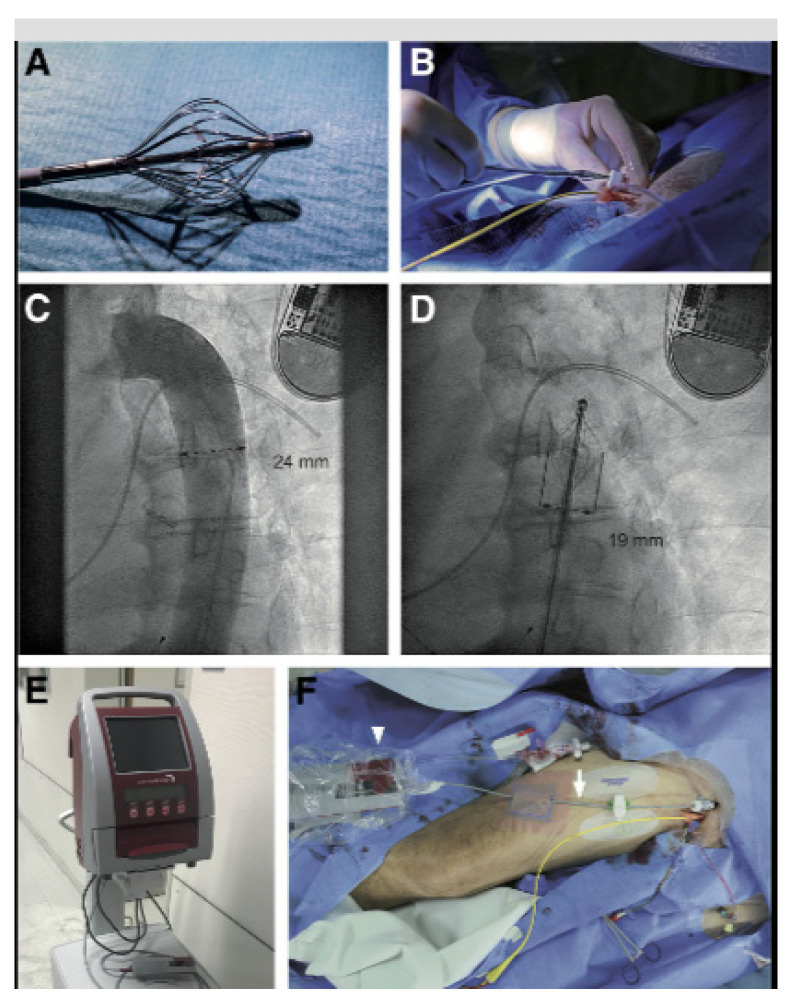
Implantation of the 10F-RCP. (**A**) RCP with unfolded cage. (**B**) Insertion through a 10F 45 cm sheath into the right common femoral artery. (**C**) Measurement of the landing zone in the descending thoracic aorta (left anterior oblique [LAO] 40° view). (**D**) Position and dimension of the unfolded pump (LAO 40° view). (**E**) Console. (**F**) Motor (arrowhead) and catheter shaft (arrow). 10F-RCP, 10F-Reitan Catheter Pump [65].

In a study by Napp et al. [65], the Reitan Catheter Pump demonstrated efficacy in a patient with advanced heart failure and CRS, showing improved renal function markers, increased GFR, and decreased levels of creatinine compared to controls, as well as a significant reduction in symptoms of fluid overload, indicating an improvement in heart and kidney function (Figure 3). The 73-year-old man had been referred for decompensated chronic heart failure (CHF) New York Heart Association (NYHA) class IV, after having experienced multiple hospital admissions for the same reason in the past. He had undergone coronary artery bypass grafting more than 20 years ago, repeated coronary intervention, ventricular tachycardia ablation, and implantation of a cardiac resynchronization defibrillator and suffered from cardiorenal syndrome. Echocardiography revealed a dilated left ventricle (LV) with a reduced ejection fraction of 30%, moderate aortic stenosis, and severe mitral and tricuspid regurgitation. The mean pulmonary arterial pressure was 37 mm Hg, the capillary wedge pressure was 18 mm Hg, and the cardiac index was 1.56 L/min/m^2^. The baseline estimated glomerular filtration rate (eGFR) was 38 mL/min/1.73 m^2^. The interdisciplinary heart team considered the patient eligible for left ventricular assist device (LVAD) implantation after appropriate cardiopulmonary recompensation and optimization of kidney function. RCP support was associated with an increase in diastolic and mean femoral arterial pressures, and urine output showed a progressive increase over time. Signs of bleeding, hemolysis, or ischemia were not observed.

#### 8.6.2. Aortix

The Aortix device is a percutaneous intra-aortic pump that is placed in the descending aorta, where it boosts forward flow to enhance systemic and renal perfusion. By drawing blood from the left ventricle and propelling it downstream in the aorta, Aortix increases renal perfusion pressure and lowers afterload, which benefits cardiac performance. This device is minimally invasive and can be deployed via catheterization, making it suitable for patients with advanced heart failure and renal impairment [41].

**Figure 4:**
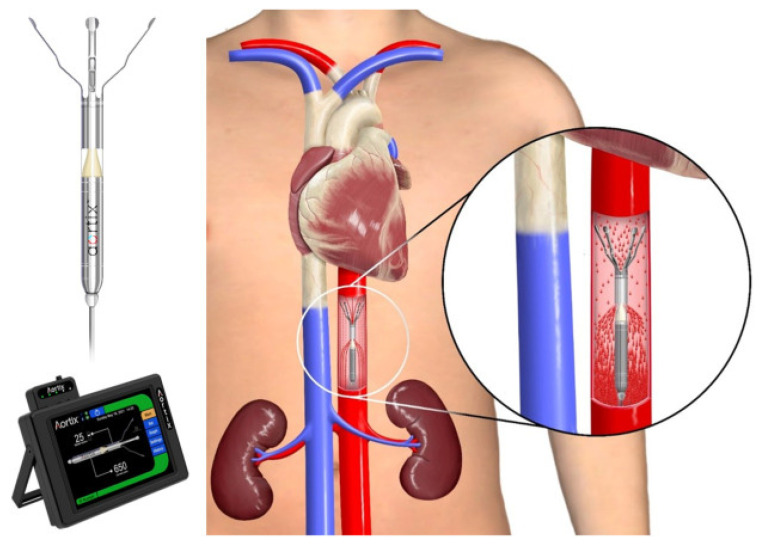
The Procyrion Aortix device is an 18F axial flow pump that is deployed in the descending aorta, positioned above the level of the renal arteries, self-centering via atraumatic nitinol struts and delivering 3.5 L/min at nominal speed (25,000 rpm). After deployment, a 6F power lead exits the femoral arteriotomy and connects to the Aortix Control System (ACS), which comprises a controller and cradle and provides hemodynamic support for CRS for up to 7 days. Once activated, a portion of native blood flow enters the pump inlet and is accelerated into high-velocity jets, which exit the pump, entraining native blood flow that passes around the pump body, resulting in increased total blood flow velocity. Aortix has been demonstrated to decrease cardiac afterload, increase cardiac output, improve renal arterial perfusion, and increase urine output [44].

Nathan et al. [44] examined the effects of Aortix in patients with CRS and found that it improved renal function within a 7-day treatment window (Figure 4). Patients receiving Aortix had a 20% increase in GFR and a corresponding decrease in serum creatinine. Additionally, Grafton et al. [40] reported that Aortix reduced the need for diuretics and improved hemodynamic stability, making it an effective option for improving renal and cardiac function in CRS patients.

The Aortix device has also been studied in an 18-patient pilot feasibility study, the results of which were recently reported [41]. Patients were, on average, 60 years old, with a median LVEF of 22.5%. Most patients (61%) were already on inotropes and had received IV diuretics (~720 mg of furosemide per day). The Aortix device was implanted in about 46 min and explanted in about 15 min. On average, CVP decreased by 39%, and PCWP by 33%. The mean peak net fluid loss was 3.6 ± 1.8 L/d. While more evidence is needed to further elucidate the risks and benefits of the Aortix MCS device, plans are underway to start the pivotal Diuretics Alone vs. Aortix Endovascular Device for AHF (DRAIN-HF) trial (NCT05677100) [36]. A planned total of 268 subjects will be randomized in a 1:1 fashion to either Aortix along with medical therapy or to medical therapy alone. The primary effectiveness endpoint is a combined composite endpoint of a clinically significant reduction in net fluid loss over 7 days and freedom from mortality or heart failure rehospitalization/therapy escalation from the baseline visit to the 30-day follow-up visit.

#### 8.6.3. ModulHeart

The ModulHeart device is a modular assist device that can be placed in either the aorta or renal arteries to boost arterial pressure and optimize blood flow to vital organs, particularly the kidneys. This device offers a unique approach by providing tailored mechanical support based on each patient’s hemodynamic profile, thereby ensuring precise control of renal perfusion pressure and minimizing risks associated with generalized arterial pressure increases.

**Figure 5:**
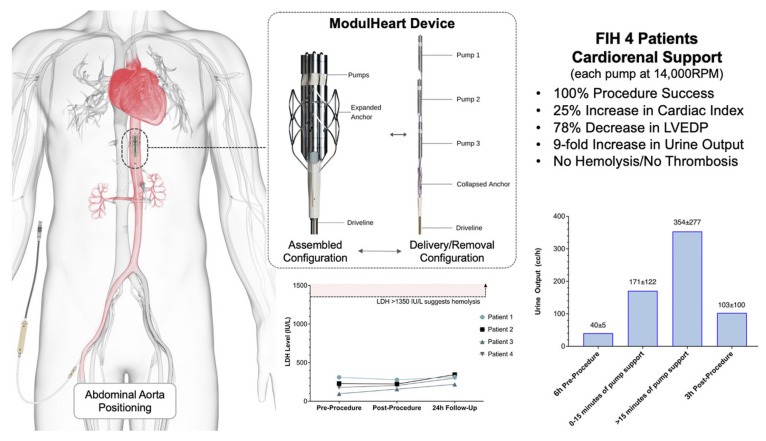
The design and results of the pilot study using ModulHeart [42].

Georges et al. [42] have shown that ModulHeart has proven useful in a study conducted in four patients undergoing a high-risk percutaneous coronary intervention (PCI), showing significant improvement in cardiac output, left ventricular end-diastolic pressure, and urine output (Figure 5). This study was a prospective, single-center, first-in-human study. The primary endpoint was procedural success, defined as successful delivery, function, and removal of the ModulHeart device. Secondary endpoints included pump hemodynamics, cardiac hemodynamics, and urine output. Four patients were enrolled and underwent high-risk PCI with ModulHeart implanted via a transfemoral approach. All four patients achieved procedural success. Under ModulHeart support, cardiac index increased by 25%, central venous pressure decreased by 37%, and left ventricular end-diastolic pressure decreased by 78%. Urine output increased by approximately nine-fold after 15 min of support. No device malfunction or procedural or device-related adverse events occurred. There was no evidence of pump thrombosis. All four patients were alive at 30 days.

This first-in-human study demonstrated the feasibility and safety of cardiorenal support with ModulHeart among patients undergoing high-risk PCI. ModulHeart demonstrated significant improvement in cardiac output, left ventricular end-diastolic pressure, and urine output. Future studies are planned to assess outcomes associated with ModulHeart support in patients with heart failure.

#### 8.6.4. Second Heart Assist

The Second Heart Assist device is an intravascular pump positioned in the aorta, where it works in synchrony with the heart’s contractions to increase forward flow and enhance arterial perfusion. This device generates pulsatile blood flow, improving renal and systemic circulation without requiring invasive surgery. Second Heart Assist is particularly beneficial for patients with a low ejection fraction, as it reduces cardiac workload while improving organ perfusion, because it can provide 72 h of uninterrupted power and is easily rechargeable, Bluetooth-enabled, and programmable to operate at as low as 2000–3000 revolutions/min as a partial support pump that can meet the individual patient’s support needs. It is intended for a wide range of long-term support durations from weeks to months or longer, such as during the vulnerable period after a HF hospitalization, as well as pretransplant support, in patients with HF and chronic kidney disease, or for ventricular recovery.

The Second Heart Assist device (Second Heart Inc., Salt Lake City, UT, USA) is a 13.5F impeller-driven pump mounted on a driveshaft placed inside a stent cage delivered percutaneously into the descending aorta only 10 cm above the renal arteries, thereby eliminating the risk of stroke. It is the most efficient pump in the field, because it operates at only 7500 revolutions/min and provides direct benefit to both the heart and kidneys. It improves cardiac output and reduces cardiac filling pressures due primarily to the afterload reduction created by activation of the impeller blades, which pull blood down through the pump, which can generate up to an additional 2.5 L of augmented pulsatile flow over native cardiac output to the kidneys and the rest of the body. The increase in renal blood flow of up to 50% over baseline can offset the intrarenal vasoconstriction associated with low cardiac output in HF, leading to increased urine output, improved kidney function, and faster decongestion. The catheter-based first-generation device can be inserted and the stent and impeller blades fully deployed in <2 min. It is designed for 24 h of support for patients admitted with acute decompensated HF who develop significant diuretic resistance. At the end of this support, the control handle can collapse the blades and stent for easy device removal of the stent and driveshaft.

**Figure 6:**
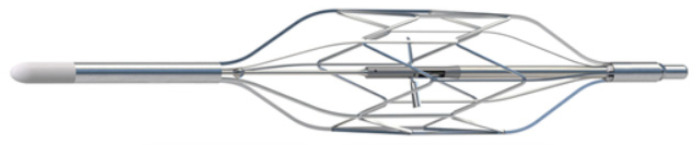
The SHA Freedom catheter is shown fully detached from delivery. The driveshaft is designed to remain in place in the descending aorta 10 cm above the renal arteries [66].

Miller et al. [66] have shown its potential as a supportive therapy for patients with advanced CRS who require enhanced renal perfusion but are at risk for worsening heart failure (Figure 6).

Second Heart Assist has also announced the successful conclusion of its second set of case studies to assess the safety and effectiveness of its Whisper percutaneous mechanical circulatory support device. These non-randomized case studies of six patients were conducted and lead by principal investigator, Adrian Ebner, head of the Cardiovascular Department at the Sanatorio Italiano Hospital in Asuncion, Paraguay, and his team [36].

### 8.7. “Fluid Shifters” in Cardio-Renal Syndrome

“Fluid shifters” are device-based therapies designed to modulate fluid balance at the level of the interstitial space, where excess fluid accumulates due to congestion and inadequate lymphatic drainage in cardio-renal syndrome (CRS). These devices target the microcirculation–interstitium–lymphatic (MIL) axis, which regulates fluid distribution in the interstitial space and plays a critical role in maintaining fluid homeostasis in CRS patients [67].

### 8.8. The Microcirculation–Interstitium–Lymphatic (MIL) Axis in Cardio-Renal Syndrome

The MIL axis is integral to the body’s fluid balance and involves complex interactions among capillaries, the interstitial space, and the lymphatic system. In healthy individuals, fluid moves across capillary walls into the interstitial space and is then absorbed by the lymphatic system, which returns it to the venous circulation. However, in CRS, increased venous pressure and reduced lymphatic clearance lead to fluid retention in the interstitial space, causing edema, tissue hypoxia, and organ congestion [68].

- Capillary Leakage: Elevated capillary pressure in heart failure increases fluid transudation into the interstitial space. This heightened capillary pressure disrupts the balance, contributing to the development of edema.
- Interstitial Fluid Accumulation: As fluid accumulates in the interstitial space, it exerts backpressure on capillaries, further impeding fluid reabsorption [69].
- Lymphatic Insufficiency: In CRS, the lymphatic system often becomes overwhelmed, failing to adequately clear excess interstitial fluid, which exacerbates congestion and worsens symptoms.

**Figure 7:**
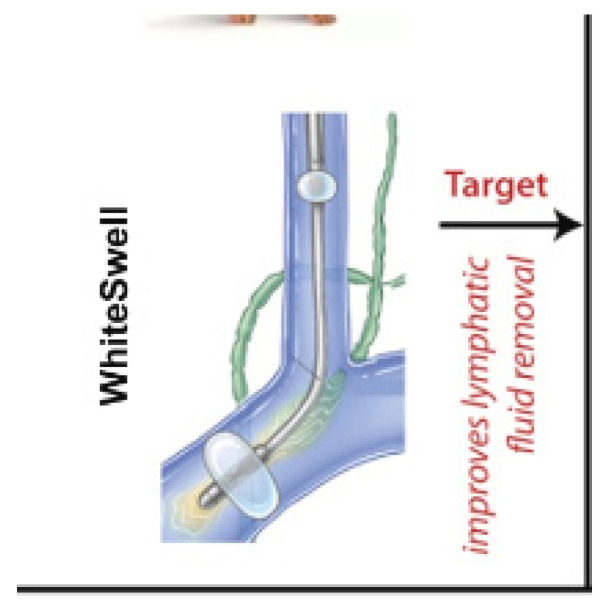
The WhiteSwell eLym device [71].

By targeting the MIL axis, “fluid shifter” devices can help alleviate interstitial edema and reduce venous congestion, thereby improving renal function and relieving symptoms in CRS patients [70] (Figure 7).

### 8.9. Device-Based Therapies for Fluid Shifting in CRS

Several devices have been developed to target the MIL axis, promoting effective fluid shifting away from the interstitial space to alleviate congestion [47].

#### 8.9.1. WhiteSwell eLym

The WhiteSwell (Galway, Ireland) eLym device is designed to enhance lymphatic clearance and reduce interstitial congestion in patients with CRS. Positioned near the thoracic duct (Figure 7), the eLym device stimulates lymphatic flow through electrical or mechanical stimulation, thereby aiding in the clearance of excess fluid from the interstitial space. By reducing lymphatic overload, eLym can alleviate venous congestion and improve renal perfusion, as shown initially in a pilot trial conducted by Abraham et al. [69], where the authors evaluated an approach to interstitial decongestion using a device to enhance lymph flow. The device was deployed in sheep with induced heart failure (HF) and acute volume overload to create a low-pressure zone at the thoracic duct outlet. Treatment decreased extravascular lung water (EVLW) volume (mL/kg) (−32% ± 9%, *p* = 0.029) compared to controls (+46% ± 9%, *p* = 0.003).

Later, Martens et al. [71] evaluated the efficacy of the WhiteSwell eLym device in patients with CRS and observed significant reductions in interstitial fluid and improvements in renal function markers, including serum creatinine and GFR. Moreover, the ongoing DELTA-HF trial [72] showed that, after only 6 months, eLym led to a reduction in tissue congestion and readmissions. In the trial, nine hospitalized patients received eLym therapy in conjunction with diuretic therapy versus six patients who received standard-of-care treatment with loop diuretics alone. The company reported that treatment with the eLym system was performed safely and successfully, with the following findings:

- The device was deployed and activated in nine patients, with a mean treatment time of 24 h.
- No patient experienced a serious procedure-, device-, or therapy-related adverse event.

Additionally, the company stated that the following early clinical results are promising and support further clinical investigation:

- Patients who underwent therapy with the eLym system plus loop diuretic lost a mean of 6.0 ± 4.6 kg from baseline to hospital discharge while maintaining kidney function, as measured by a stable or improved creatinine (mean Δ −0.10 ± 0.12 mg/dL).
- The loop diuretic-only group lost a mean of 3.3 ± 3.7 kg.
- One of the nine treated patients (11%) was hospitalized within 30 days of discharge.

#### 8.9.2. AquaPASS

**Figure 8:**
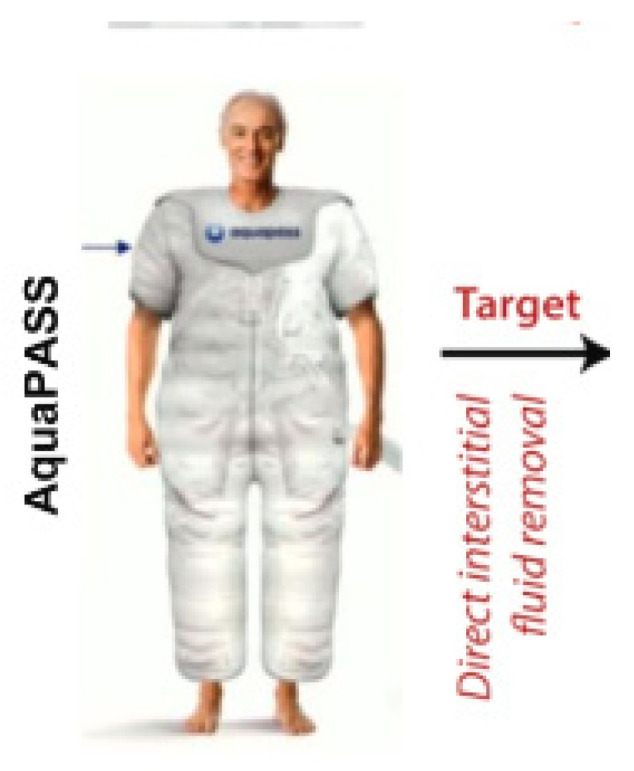
The AquaPASS device [71].

The AquaPASS device leverages peritoneal fluid absorption to reduce central and venous pressures (Figure 8). When the device is placed in the peritoneal cavity, AquaPASS promotes the translocation of excess fluid from the blood into the peritoneal space, where it can be absorbed and eventually eliminated. This process, similar to peritoneal dialysis, facilitates a gradual shift of fluid away from the circulatory system and alleviates the burden of excess volume on the heart and kidneys.

The AquaPASS system has been tested in CRS patients who have diuretic resistance [36] and is expected to have promising results in terms of decongestion and survival rate. Aronson et al. [73] used this device designed to enhance fluid and salt loss via the eccrine sweat glands. Skin temperature in the lower body increased from 35 °C to 38 °C, where the slope of the relationship between temperature and sweat production was linear. The sweat evaporates instantaneously, thus avoiding awareness of perspiration. The primary efficacy endpoint was the ability to increase skin temperature to the desired range. A secondary efficacy endpoint was a clinically meaningful hourly sweat output, defined as ≥150 mL/h. The primary safety endpoint was any procedure-related adverse events.

Six normal subjects and eighteen patients with congestion were studied. Participants underwent three treatment sessions of up to 4 h. Skin temperature increased to a median of 37.5 °C (interquartile range, 37.1–37.9 °C), with the median core temperature increasing by 0.2 °C (interquartile range, 0.1–0.3 °C). The median hourly weight loss during treatment was 215 g/h (interquartile range, 165–285; range, 100–344 g/h). In 80% of treatment procedures, the average sweat rate was ≥150 mL/h. There were no significant changes in hemodynamic variables or renal function and no procedure-related adverse events.

#### 8.9.3. Reprieve

**Figure 9:**
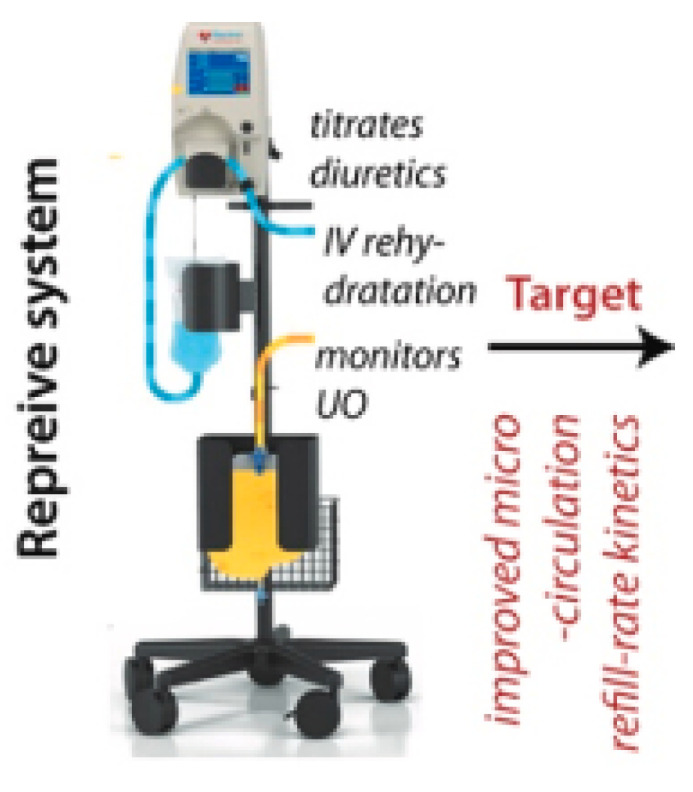
The Reprieve device [44].

The Reprieve device (Figure 9) works by modulating venous return and reducing fluid retention in the interstitial spaces. Implanted subcutaneously, Reprieve gently manipulates fluid levels by creating mild suction forces to draw interstitial fluid into lymphatic channels, enhancing its return to the central circulation and preventing tissue edema [74]. This method effectively lowers CVP and improves systemic circulation by redistributing fluid from the interstitial space.

Studies TARGET-1 and TARGET-2 comprised 19 patients hospitalized with AHF (mean age 67 ± 10 years, 18 male, ejection fraction 34 ± 15%, median N-terminal pro-B-type natriuretic peptide 4492 pg/mL). Patients served as their own controls: each patient underwent 24 h of standard diuretic therapy followed by 24 h of diuretics with Reprieve therapy (with normal saline used for matched volume replacement). The primary efficacy endpoint of actual fluid loss not exceeding the target fluid loss at the end of therapy was met in all 19 (100%) patients. The mean diuresis during Reprieve therapy was 6284 ± 2679 mL (vs. 1966 ± 1057 mL 24 h before therapy) and 2053 ± 888 mL (24 h after therapy) (both *p* < 0.0001). At the end of therapy, patient global assessment improved from 7.7 ± 1.1 to 3.0 ± 1.3 points (*p* < 0.001), central venous pressure decreased from 15.5 ± 5.3 mmHg to 12.8 ± 4.8 mmHg (*p* < 0.05), and the median urine sodium loss was 9.7 mmol/h [1,2,3,4,5,6,7,8,42,44,66]. Reprieve therapy was safe, systolic blood pressure remained stable, mean creatinine dropped from 1.45 ± 0.4 mg/dL to 1.26 ± 0.4 mg/dL (*p* < 0.001), and biomarkers of renal injury did not change during treatment.

#### 8.9.4. DSR 2.0 (Direct Sodium Removal)

**Figure 10:**
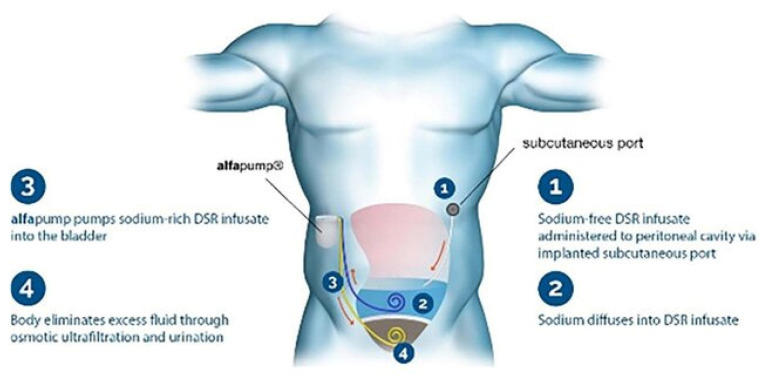
DSR 2.0 device [6].

DSR 2.0 is an innovative therapy that focuses on fluid removal by targeting sodium directly, facilitating the osmotic mobilization of fluid out of the interstitial space. DSR 2.0 is administered through a peritoneal catheter, where it draws sodium and fluid from the interstitial compartment into the peritoneal cavity, effectively reducing both sodium levels and fluid volume. This technique not only alleviates congestion but also improves hemodynamic stability by lowering CVP and supporting kidney function (Figure 10).

The recent data on the AlfaPump^®^ (DSR device developed by Sequana Medical N.V., Sint-Denijs-Westrem, Belgium) from RED DESERT [36] (eight euvolemic patients) and SAHARA [36] (ten hypervolemic patients), in which patients with HF requiring high doses of loop diuretics were withdrawn from diuretic medication and serial DSR was utilized to achieve and maintain euvolemia, showed that, at 1-year follow-up, the necessary diuretic dose remained substantially below baseline (30 [IQR 7.5–40] mg furosemide equivalents/day). Moreover, multiple dimensions of kidney function such as filtration, uremic toxin excretion, kidney injury, and electrolyte handling, improved (*p* < 0.05 for all). HF-related biomarkers including N-terminal pro-B-type natriuretic peptide, carbohydrate antigen-125, soluble ST2, interleukin-6, and growth differentiation factor-15 (*p* < 0.003 for all) also improved.

This goes to show that, in patients with HF and diuretic resistance, serial DSR therapy with loop diuretic withdrawal is feasible and associated with substantial and persistent improvement in diuretic resistance and several cardiorenal parameters. If replicated in randomized controlled studies, DSR may represent a novel therapy for diuretic resistance and CRS [75].

### 8.10. Are These Studies Really Useful? How? When? Can We Make Them Better?

Device-based therapies for cardio-renal syndrome (CRS) represent a promising frontier in managing the complex interplay between heart and kidney dysfunction. However, as the field advances, it has become increasingly evident that traditional endpoints in clinical trials may not fully capture the therapeutic impact and long-term benefits of these devices. The limitations of current trial endpoints are the need for more comprehensive and relevant measures and the future potential and challenges of these devices in CRS treatment.

From a medical standpoint, endpoints in clinical trials should fulfill several key criteria: they must occur frequently enough to be measurable, be easy to assess with reliability, respond to the intervention being studied, and hold significance for all stakeholders—patients, healthcare providers, industry, and payers. However, these groups often prioritize different outcomes. Physicians typically aim to reduce mortality, payers focus on reducing readmissions, patients prioritize improved quality of life (measured through patient-reported outcomes), and industry seeks to demonstrate safety and efficacy, often using surrogate endpoints to facilitate early market access.

To address these varying priorities, approaches like combined endpoints or analytical frameworks such as the win ratio (which prioritizes fatal outcomes over nonfatal ones) allow the inclusion of multiple relevant clinical measures. These methods are particularly useful in pivotal trials, which are large-scale studies designed to support regulatory approval. Such trials require substantial patient populations and financial investment, which can delay their completion and integration into clinical practice, especially given the high costs of many of the devices discussed. A critical consideration is determining which intermediate endpoints, such as physiological effects observed in pilot studies for acute CRS, can reliably predict success in pivotal trials using clinical outcomes like mortality or hospitalization.

In addition to the current scarcity of data supporting the effectiveness of these devices, several challenges and uncertainties remain. One significant issue is the lack of clear guidance on when to conclude decongestion therapy or how to define complete decongestion at discharge. Efforts to define “euvolemia” in heart failure have been inconsistently applied in practice and trials. Furthermore, the concept of euvolemia, which is volume-focused, does not adequately reflect the absence of congestion, a pressure-related phenomenon, due to weak correlations among intravascular volume, extravascular volume, and pressure.

Another challenge is that, while short-term device use can improve hemodynamic parameters, the potential for congestion rebound underscores the importance of integrating devices into a comprehensive treatment strategy.

Lastly, there is no robust evidence yet that short-term interventions, whether drug- or device-based, during acute decompensated heart failure (ADHF) episodes lead to sustained improvements in outcomes after discharge. Although early human studies have shown promising effects on intermediate outcomes such as hemodynamic stability, urine output, and sodium excretion, only randomized controlled trials can provide definitive evidence of efficacy. Single-arm or open-label studies are prone to biases, including regression to the mean, which limits their reliability.

### 8.11. Limitations of Current Trial Endpoints in CRS

Most clinical trials for CRS device therapies have relied on standard endpoints commonly used in heart failure or kidney disease studies, such as:

- Changes in Creatinine Levels: While serum creatinine is an indicator of kidney function, it is affected by many factors beyond actual renal health, including body mass, hydration status, and medications. Moreover, short-term fluctuations in creatinine do not necessarily reflect long-term kidney health.
- Glomerular Filtration Rate (GFR): GFR is another widely used measure, yet it can remain stable or even improve transiently without signifying sustained renal recovery.
- Hospitalization Rates: While a reduction in hospitalizations can indicate improved outcomes, it is also influenced by factors such as local healthcare policies and patient adherence, making it an indirect measure of device effectiveness.
- Mortality: Although mortality is a critical endpoint, it is a relatively coarse measure that fails to capture the nuanced benefits of improved quality of life or symptom relief that many device therapies offer in CRS.

These endpoints may miss improvements in hemodynamic parameters, venous pressure, or other physiological metrics that directly correlate with CRS’s pathophysiology. Without refined endpoints that capture these nuances, assessing the true efficacy of novel device-based therapies remains challenging.

### 8.12. Suggested Novel Endpoints for CRS Trials

To better assess the efficacy of device-based therapies in CRS, more relevant and specific endpoints should be considered. These include:

- Central Venous Pressure (CVP): CVP directly reflects venous congestion, a core pathophysiological component in CRS, particularly in “backward failure” scenarios. Monitoring CVP changes could provide direct insight into the effectiveness of “puller” devices such as the Doraya catheter.

One easily reproducible and reliable method to assess venous congestion in critical situations, and not only these situations, is by using the VExUS [76] protocol of echography. It relies on measurements of inferior vena cava (IVC) size and Doppler assessments of the hepatic vein (HV), portal vein (PV), and intrarenal vein, thereby providing real-time insights into hemodynamic status and guiding therapeutic interventions. The VExUS score is valuable in assessing and predicting venous congestion, especially regarding AKI prediction risk and guiding interventions. However, its utility in predicting outcomes in acute heart failure patients appears less certain [77].

- Cardiac Output and Renal Perfusion Pressure: Measuring renal perfusion directly can provide a clearer picture of how “pushers” like Aortix and ModulHeart are improving renal function by enhancing forward flow, which is often inadequately represented by traditional endpoints.
- Quality of Life Metrics: Patient-reported outcomes that measure symptom relief, exercise tolerance, and daily functioning should be prioritized, as these reflect real-world benefits and may correlate better with the overall impact of therapy.

### 8.13. Challenges and Uncertainties to Overcome

While device-based therapies are promising, there are significant challenges that need to be addressed to ensure their widespread use and efficacy in CRS management:

- Complexity of CRS Pathophysiology: The mechanisms driving CRS are multifaceted, involving interactions among cardiac output, venous congestion, neurohormonal activation, and more. Determining the optimal device for each patient based on individual pathophysiology requires sophisticated diagnostic tools and experience.
- Patient Selection: The heterogeneity of CRS poses a challenge for patient selection. Identifying which patients will benefit most from “pullers”, “pushers”, or “fluid shifters” requires precise criteria and biomarkers, which are still under development.
- Safety and Long-Term Data: Most device trials have focused on short-term efficacy. The long-term safety of devices like the Doraya catheter, Aortix, and WhiteSwell eLym needs to be assessed, particularly regarding risks of thrombosis, infection, and device durability. Ongoing studies like MOJAVE [36] are gathering data but may take years to yield comprehensive results.
- Integration with Existing Care Models: Incorporating device-based therapies into current heart failure and nephrology care pathways requires collaboration across specialties, which can be logistically challenging. Additionally, many healthcare systems are not yet equipped to handle the operational demands of these devices.
- Cost-Effectiveness: Device-based therapies are generally more expensive than pharmacologic treatments. Demonstrating cost-effectiveness through reductions in hospitalizations, emergency visits, and long-term health improvements will be crucial for their adoption.

## 9. Conclusions

The development of device-based therapies for CRS has the potential to shift the treatment paradigm from reactive to proactive management. By refining trial endpoints to reflect true improvements in CRS pathophysiology, and by addressing the logistical, clinical, and financial challenges of these devices, we may unlock their full potential to deliver more effective, targeted treatment for this complex syndrome. Future research that includes comprehensive endpoints and long-term data, as well as thoughtful patient selection and cross-specialty collaboration, will be instrumental in advancing device-based therapies as a cornerstone in CRS management.

## Figures and Tables

**Table 1:** DRI2P2S Classifications and Examples [6].

Category	Mechanism	Examples
Decongestive (D) or Dilators (D)	Reduce fluid overload and congestion Improve blood flow, reduce vascular resistance	Loop diuretics (furosemide), aquapheresis Splanchnic nerve blockade
Renal Replacement (R)	Provides artificial renal function	CRRT, intermittent hemodialysis
Inotropes (I)	Enhance cardiac contractility	Dobutamine, Levosimendan
Interstitial (Fluid Management) (I)	Manages interstitial fluid	WhiteSwell eLym, AquaPASS
Pullers (P)	Reduce venous congestion	Doraya catheter, preCARDIA system
Pushers (P)	Enhance arterial flow and renal perfusion	Aortix, ModulHeart
Sympatholytics (S)	Inhibit SNS activity to lower vascular resistance	Beta-blockers
